# Antioxidant and Antibacterial Activities of Secondary Metabolites Produced by *Streptomyces* Isolates Against Extended‐Spectrum β‐Lactamase‐Producing Bacteria

**DOI:** 10.1002/mbo3.70282

**Published:** 2026-04-19

**Authors:** Abdirasak Sharif Ali, Yahye Ahmed Nageye, Kizito Eneye Bello

**Affiliations:** ^1^ Faculty of Medicine and Health Sciences SIMAD University Mogadishu Somalia; ^2^ Department of Microbiology, Faculty of Natural Science Kogi State (Prince Abubakar Audu) University, Anyigba Anyigba Kogi State Nigeria

**Keywords:** antibacterial activity, antioxidant activity, ESBL‐producing bacteria, molecular docking, *Streptomyces* secondary metabolites

## Abstract

Extended‐spectrum β‐lactamase (ESBL)–producing Gram‐negative bacteria pose a severe therapeutic challenge globally. *Streptomyces* remain one of the most prolific natural sources of antibacterial and antioxidant secondary metabolites, yet their activity against ESBL‐producing pathogens remains under‐explored. Soil‐derived *Streptomyces* isolates were screened for bioactivity, and the most potent strain (SM7) was identified by 16S rRNA sequencing. Secondary metabolites were extracted using ethyl‐acetate and evaluated for antibacterial activity against ESBL‐producing *Escherichia coli*, *Klebsiella pneumoniae*, and *Enterobacter cloacae* using agar diffusion, MIC, and MBC assays. Antioxidant activity was assessed using DPPH and ABTS assays, while GC–MS and molecular docking were employed to identify and characterize bioactive compounds. *Streptomyces sp*. SM7 exhibited strong antibacterial activity, producing inhibition zones of 21.4 ± 0.6 mm, 19.2 ± 0.4 mm, and 17.6 ± 0.5 mm against ESBL‐producing *E. coli*, *K. pneumoniae*, and *E. cloacae*, respectively. MIC values ranged from 62.5 to 250 µg/mL, with bactericidal MBC/MIC ratios of 2. The extract showed potent antioxidant activity with DPPH and ABTS IC₅₀ values of 48.9 µg/mL and 61.4 µg/mL, respectively. GC–MS identified 18 bioactive compounds, with 2,4‐di‐tert‐butylphenol (18.6%) as the major constituent, which exhibited a docking affinity of −7.1 kcal/mol against bacterial DHFR. *Streptomyces sp*. SM7 produces phenolic‐ and fatty‐acid‐rich metabolites with potent bactericidal and antioxidant activities against ESBL‐producing pathogens, highlighting its promise as a natural source of next‐generation antimicrobial agents. These findings support *Streptomyces* sp. SM7 as a promising lead for downstream purification, mechanism‐guided optimization, and future drug‐development efforts targeting difficult‐to‐treat ESBL‐producing Enterobacterales.

## Graphical abstract

soil‐derived *Streptomyces* sp. SM7 with bactericidal and antioxidant activities

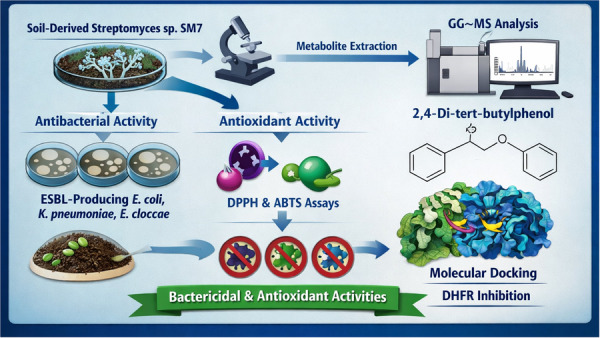

## 1 Introduction

The swift rise and worldwide spread of antimicrobial resistance (AMR) constitute one of the gravest threats to public health in the 21st century. Extended‐spectrum β‐lactamase (ESBL)‐producing bacteria represent a significant challenge among resistant pathogens due to their capacity to hydrolyze a wide array of β‐lactam antibiotics, including third‐generation cephalosporins and monobactams (Saliba et al. 2023). ESBL‐producing *Escherichia coli, Klebsiella pneumoniae, Pseudomonas aeruginosa*, and analogous Gram‐negative pathogens are progressively linked to hospital and community‐acquired infections, leading to extended hospitalization, heightened mortality, and rising healthcare expenditures globally (Jorgensen et al. 2023; Kizito 2013).

The impact of ESBL‐mediated resistance is particularly severe in low‐ and middle‐income nations, where antibiotic misuse, insufficient infection control, and restricted access to advanced therapies intensify the proliferation of resistant strains (Guehria et al. 2025). The associated morbidity of drug resistance is higher in immunocompromised patients and during pregnancy, as well as the rate of mutation in some pathogen that eventually degenerates into mutations and its associated drug resistance (Jorgensen et al. 2023). Traditional treatment modalities are swiftly diminishing in effectiveness, and the pipeline for new antibiotics is notably inadequate. This crisis highlights the pressing necessity for alternative antimicrobial agents with innovative mechanisms of action capable of effectively targeting multidrug‐resistant bacteria.

Historically, natural products have been the cornerstone of antibiotic discovery, with more than two‐thirds of clinically utilized antibiotics originating from microbial sources (Kizito 2013). The genus *Streptomyces* is notably prolific, contributing around 70% of naturally derived antibiotics, such as streptomycin, tetracycline, chloramphenicol, and erythromycin. *Streptomyces* species are filamentous, Gram‐positive actinobacteria prevalent in soil and various ecological environments, known for their extensive production of secondary metabolites exhibiting antibacterial, antifungal, antiviral, anticancer, and antioxidant properties (Guehria et al. 2025).

*Streptomyces* generate structurally diverse and biologically active secondary metabolites, including polyketides, non‐ribosomal peptides, aminoglycosides, macrolides, phenazines, and alkaloids (Sanjivkumar et al. 2016). These compounds frequently demonstrate distinct mechanisms of action, including the inhibition of protein synthesis, disruption of cell membrane integrity, interference with DNA replication, and modulation of redox homeostasis. Significantly, numerous metabolites derived from *Streptomyces* remain unexamined due to silent biosynthetic gene clusters that are activated solely under environmental or cultivation conditions, underscoring the genus as an ongoing source for novel antimicrobial discovery (Abdulkareem et al. 2024).

Recent years have witnessed a resurgence of interest in investigating *Streptomyces* metabolites as potential agents against multidrug‐resistant and ESBL‐producing bacteria (Guehria et al. 2025; Khaledi and Obeid 2025). In contrast to traditional β‐lactam antibiotics, numerous secondary metabolites function independently of β‐lactam targets. They can circumvent prevalent resistance mechanisms, including enzymatic degradation or modifications to penicillin‐binding proteins. *Streptomyces*‐derived compounds are promising candidates for addressing ESBL‐associated infections.

In addition to antibacterial activity, oxidative stress is pivotal in microbial pathogenesis and host immune responses. Reactive oxygen species (ROS) are produced during infection and inflammation, aiding in bacterial eradication; however, excessive oxidative stress can harm host tissues and intensify disease severity. Antioxidant compounds can regulate redox equilibrium, diminish inflammation, and bolster host resilience during infection. Several secondary metabolites of *Streptomyces* exhibit potent antioxidant properties, including phenazine derivatives, flavonoid‐like compounds, and quinones, which can scavenge free radicals and chelating metal ions (Monciardini et al. 2002; Raheem and Abdalshheed 2024).

The combined antibacterial and antioxidant properties of *Streptomyces* metabolites are increasingly acknowledged as synergistic qualities that may improve therapeutic effectiveness. Antioxidant activity may safeguard host tissues, whereas antibacterial compounds directly impede pathogen proliferation, providing a comprehensive strategy for infection management. Furthermore, the modulation of oxidative stress has been demonstrated to affect bacterial susceptibility to antimicrobials, indicating that antioxidant‐active metabolites may indirectly enhance antibacterial efficacy against resistant pathogens.

Despite comprehensive research on *Streptomyces* as antibiotic producers, limited studies have systematically assessed the concurrent antioxidant and antibacterial properties of *Streptomyces* secondary metabolites against ESBL‐producing bacteria. Most existing studies concentrate on non‐resistant clinical strains or isolated bioactivity endpoints, resulting in a significant knowledge deficit concerning the potential of these metabolites to tackle contemporary resistance issues.

The increasing prevalence of ESBL‐producing pathogens necessitates the investigation of sustainable and effective alternative antimicrobial strategies that can surmount resistance mechanisms. Examining *Streptomyces* isolates from various ecological environments may uncover unique metabolite profiles with increased efficacy against resistant bacteria. These studies not only facilitate antibiotic discovery but also elucidate the environmental and biochemical factors influencing secondary metabolite biosynthesis.

This study seeks to isolate and characterize *Streptomyces* strains and assess the antioxidant and antibacterial properties of their secondary metabolites against ESBL‐producing bacterial pathogens.

## 2 Materials and Methods

### 2.1 Sample Collection and Isolation of *Streptomyces*

Soil samples were aseptically collected from undisturbed environments at a depth of 10–15 cm and transported to the laboratory in sterile polyethylene bags. Samples were air‐dried at ambient temperature for 5–7 days to inhibit the proliferation of rapidly growing bacteria.

Ten grams of each soil sample were suspended in 90 mL of sterile physiological saline and serially diluted to 10⁻⁶. Aliquots (0.1 mL) of suitable dilutions were applied to Starch Casein Agar (SCA) augmented with nystatin (50 µg/mL) and nalidixic acid (20 µg/mL) to suppress fungal and Gram‐negative bacterial proliferation. Plates were incubated at 28°C ± 2°C for a duration of 7 to 14 days.

Presumptive *Streptomyces* colonies were chosen based on their powdery appearance, formation of aerial mycelia, earthy Oduor, and pigmentation, and were subsequently sub‐cultured multiple times to achieve pure isolates (Bello et al. 2024).

### 2.2 Phenotypic and Molecular Identification of *Streptomyces* Isolates

Morphological characterization was conducted via light microscopy, after Gram stain. Standard methods were employed to perform biochemical tests, including catalase activity, starch hydrolysis, casein hydrolysis, and the utilization of specific carbon sources.

Genomic DNA was extracted from selected isolates for molecular identification using a commercial bacterial DNA extraction kit. The 16S rRNA gene was amplified with universal primers (SM‐Forward: 5′‐GGTGGCGAAGGCGGA‐3′, and SM‐reversed: 5′‐GAACTGAGACCGGCTTTTTGA‐3′) (Monciardini et al. 2002). PCR products were sequenced and analyzed against reference sequences in the NCBI GenBank database utilizing BLAST. Phylogenetic analysis was conducted using MEGA software.

### 2.3 Production and Extraction of Secondary Metabolites

Actively proliferating *Streptomyces* isolates were inoculated into ISP‐2 broth and incubated at 28°C for 7 to 10 days under shaking conditions at 150 rpm Following fermentation, cultures underwent centrifugation at 8000 rpm for 15 min to segregate biomass from the supernatant. During preliminary screening, crude extracts from all eight presumptive *Streptomyces* isolates were compared against the same ESBL‐producing test panel, and isolate SM7 consistently produced the largest inhibition zones (Table S1).

The supernatant was extracted twice with an equivalent volume of ethyl acetate, utilizing a separatory funnel. The organic phase was concentrated under reduced pressure with a rotary evaporator to yield crude secondary metabolite extracts. Extracts were preserved at 4°C pending subsequent analysis (Bello et al. 2024).

### 2.4 Test Bacterial Isolates

Clinical isolates of ESBL‐producing *E. coli, K. pneumoniae*, and *Enterobacter species* were acquired from a tertiary healthcare institution. Bacterial identification was validated through standard biochemical assays. ESBL production was phenotypically validated through the double‐disc synergy test (DDST) utilizing ceftazidime, cefotaxime, and amoxicillin–clavulanic acid discs in accordance with CLSI guidelines. All clinical ESBL isolates were obtained through routine laboratory collections using institutionally approved procedures, and no patient identifiers or traceable clinical metadata were retained at any stage of the study.

### 2.5 Antibacterial Activity Assay

The antibacterial efficacy of crude extracts from *Streptomyces* was assessed utilizing the agar well diffusion technique. Mueller–Hinton agar plates were inoculated with standardized bacterial suspensions calibrated to the 0.5 McFarland standard.

Wells with a diameter of 6 mm were drilled into the agar, and 100 µL of each extract at different concentrations was added. Plates were incubated at 37°C for 24 h, after which the zones of inhibition were measured in millimeters. Ciprofloxacin functioned as the positive control, whereas the solvent acted as the negative control (Juwitaningsih et al. 2020).

### 2.6 Determination of MIC and MBC

The minimum inhibitory concentration (MIC) and minimum bactericidal concentration (MBC) were ascertained utilizing the broth microdilution technique in 96‐well microtiter plates. Serial twofold dilutions of the extracts were conducted in Mueller–Hinton broth.

After incubation at 37°C for 24 h, the minimum inhibitory concentration (MIC) was determined as the lowest concentration exhibiting no visible growth. Aliquots from wells exhibiting no growth were subculture onto nutrient agar plates to ascertain the minimum bactericidal concentration (MBC) (Alissa 2025). MBC was defined as the lowest extract concentration producing ≥ 99.9% killing of the initial inoculum after subculture onto extract‐free agar, and MBC/MIC ratios ≤ 4 were interpreted as bactericidal activity (Pankey and Sabath 2004).

### 2.7 Antioxidant Activity Assays

#### 2.7.1 DPPH Radical Scavenging Assay

The extracts' free radical scavenging activity was evaluated using the DPPH assay. In summary, 1 mL of 0.1 mM DPPH solution was combined with 1 mL of extract at varying concentrations. The solution was incubated in darkness for 30 min, and absorbance was recorded at 517 nm. Ascorbic acid served as the reference standard (Tatke et al. 2018).

#### 2.7.2 ABTS Radical Cation Decolorization Assay

The ABTS radical scavenging activity was assessed by reacting extracts with a pre‐formed ABTS•⁺ solution. Absorbance was quantified at 734 nm, and the results were articulated as percentage inhibition (Thaipong et al. 2006).

### 2.8 Chemical Profiling of Bioactive Extracts

The most active extracts underwent GC–MS analysis. Separation was accomplished utilizing a capillary column, and compounds were identified by correlating mass spectra with those in the NIST library. Identified compounds were classified according to their chemical categories and documented biological activities (Thamer and Thamer 2023). Relative abundance values were calculated by peak‐area normalization of the total ion chromatogram rather than by external calibration. Compound identities should therefore be regarded as tentative because annotation was based on NIST library matching; authentic analytical standards were not used for confirmation, which represents a limitation of the present chemical profiling. GC–MS analysis of the most active extract was performed on a Shimadzu GCMS‐QP2010 equipped with an Rtx‐5MS capillary column (30 m × 0.25 mm id, 0.25 µm film thickness). Helium was used as the carrier gas at a constant flow rate of 1.41 mL/min. A 1 µL aliquot of extract was injected at an injector temperature of 250°C in split mode (1:15). The oven temperature was programmed from 50°C (held 2 min) to 300°C at 5°C/min, with a final hold of 5 min. Mass spectra were acquired in electron ionization mode at 70 eV, with ion source and interface temperatures of 200°C and 280°C, respectively, over a scan range of m/z 35–500.

### 2.9 Molecular Docking Analysis

#### 2.9.1 Selection and Preparation of Target Proteins

To examine the potential molecular mechanisms responsible for the antibacterial efficacy of *Streptomyces*‐derived secondary metabolites against ESBL‐producing bacteria, critical bacterial target proteins linked to cell wall synthesis and DNA replication were identified. These comprised DNA gyrase (GyrA), dihydrofolate reductase (DHFR), and penicillin‐binding protein (PBP) (Hooper and Jacoby 2016; Sauvage et al. 2008).

The three‐dimensional crystal structures of the target proteins were obtained from the Protein Data Bank (PDB). Structures were chosen based on their high resolution and significance to Gram‐negative bacteria. Before docking, protein structures were prepared by eliminating water molecules, heteroatoms, and co‐crystallized ligands. Polar hydrogens were incorporated, and Kollman charges were allocated utilizing AutoDock Tools (ADT) (Trott and Olson 2010).

#### 2.9.2 Ligand Preparation

Bioactive compounds identified through GC–MS analysis of the *Streptomyces* crude extracts were chosen as ligands for docking analysis. The chemical structures of the desired compounds were obtained from the PubChem database in SDF format and transformed into PDB format utilizing Open Babel (O'boyle et al. 2011). Among the detected constituents, 2,4‐di‐tert‐butylphenol was prioritized for docking because it was the most abundant phenolic compound, has a compact scaffold compatible with enzyme active sites, possesses a hydrogen‐bond‐donating hydroxyl group, and has prior literature support for antibacterial activity (Seenivasan et al. 2022). By contrast, the major fatty‐acid derivative hexadecanoic acid methyl ester was retained in the discussion as a potentially contributory membrane‐active metabolite, but it was not prioritized for the present docking workflow because long‐chain hydrophobic esters generally yield less target‐specific interaction hypotheses in rigid receptor docking.

Ligands underwent energy minimization utilizing the MMFF94 force field, and Gasteiger charges were allocated. Rotatable bonds were delineated, and ligand structures were archived in PDBQT format for docking purposes (Meng et al. 2011).

#### 2.9.3 Docking Procedure

Molecular docking was conducted with Auto Dock Vina to ascertain the binding affinities and interaction patterns between chosen *Streptomyces*‐derived compounds and the target proteins. Grid boxes were delineated to encompass the active sites of each protein, based on established ligand‐binding residues (Trott and Olson 2010; Meng et al. 2011). Molecular docking was performed using AutoDock Vina. The grid boxes were centered on the native ligand binding pockets identified from the crystal structures. The grid center coordinates and box sizes were defined as follows: 1KZN (*X* = 24.56, *Y *= −5.32, *Z* = 37.89; box = 22 × 22 × 22 Å); 1RX2 (*X* = 9.74, *Y* = −14.56, *Z* = 27.31; box = 20 × 20 × 20 Å); and 6RKS (*X* = 16.21, *Y* = 45.38, *Z* = −9.62; box = 24 × 24 × 24 Å). The search space encompassed the active‐site residues surrounding the co‐crystallized ligands.

The docking parameters were configured to default exhaustiveness values, resulting in the generation of multiple docking poses for each Ligand–protein complex. The binding affinity (measured in kcal/mol) of the highest‐ranked pose was documented for subsequent analysis (Laskowski and Swindells 2011).

#### 2.9.4 Visualization and Interaction Analysis

The docked complexes were examined utilizing PyMOL and Discovery Studio Visualiser. Protein–ligand interactions, such as hydrogen bonds, hydrophobic interactions, π–π stacking, and van der Waals forces, were examined and correlated with crucial amino acid residues implicated in ligand binding (Morris et al. 2009).

#### 2.9.5 Validation of Docking Protocol

To validate the docking protocol, co‐crystallized ligands, when available, were re‐docked into their corresponding binding sites, and the resultant poses were compared with the original crystal structures. A root mean square deviation (RMSD ≤ 2.0 Å) was deemed acceptable and indicative of dependable docking precision (Trott and Olson 2010).

#### 2.9.6 Comparative Docking With Standard Antibiotics

For comparative analysis, standard antibiotics frequently employed against Gram‐negative bacteria, such as ciprofloxacin (a DNA gyrase inhibitor) and trimethoprim (a DHFR inhibitor), were docked to the same targets under uniform conditions. The binding affinities and interaction profiles were contrasted with those of the compounds derived from *Streptomyces* (Sköld 2000).

### 2.10 Statistical Analysis

All experiments were conducted in triplicate. Data were presented as mean ± standard deviation (SD). Statistical significance was assessed using one‐way ANOVA, with *p* < 0.05 deemed statistically significant. Where relevant, one‐way ANOVA was followed by comparison of each treatment group with the positive control using Dunnett‐type post hoc testing; exact *p*‐values are now provided in the revised activity tables.

## 3 Results

### 3.1 Isolation and Identification of *Streptomyces* Isolate

**Figure 1:**
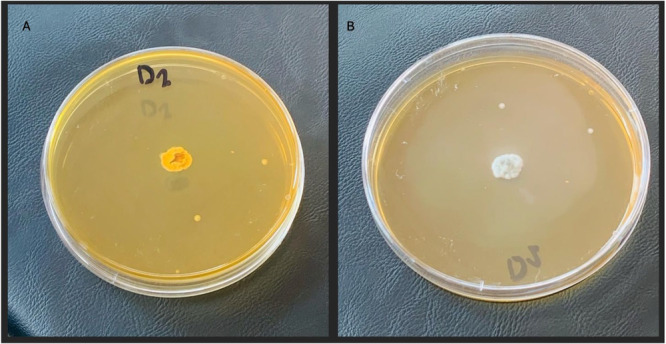
Cultural morphology of *Streptomyces* sp. SM7. (A) Substrate mycelium of *Streptomyces* sp. SM7. (B) Aerial mycelium of *Streptomyces* sp. SM7.

A total of eight presumptive *Streptomyces* isolates were initially obtained from soil samples. After initial screening for antibacterial efficacy against ESBL‐producing bacteria, one isolate (designated *Streptomyces* sp. SM7) (Figure 1) exhibited significantly enhanced bioactivity and was chosen for further examination (Table S1).

Morphologically, isolate SM7 displayed well‐developed aerial and substrate mycelia (Figure 1) with a chalky white appearance and emitted an earthy odour typical of *Streptomyces* species. The isolate was Gram‐positive and exhibited extensively branched filamentous structures under light microscopy.

**Figure 2:**
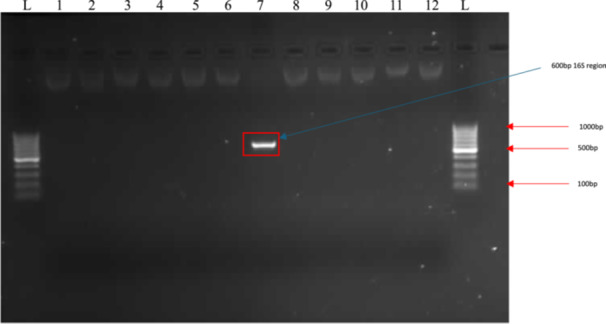
A gel image for the PCR assay specificity for detecting and amplifying the 16S region of *Streptomyces* sp. L: 100 base pair ladder.

**Figure 3:**
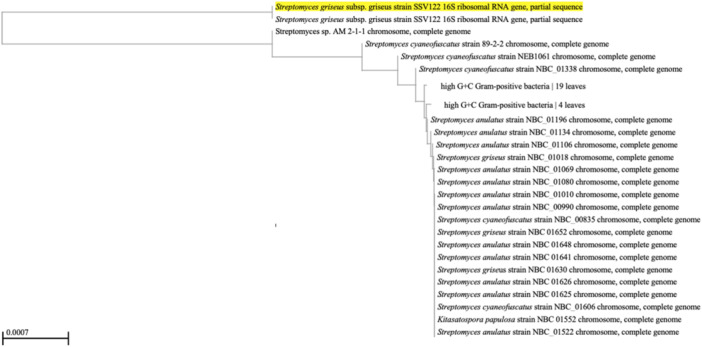
Phylogenetic tree showing the ancestral relatedness of *Streptomyces* isolate SM7 (*Streptomyces griseus*). The organism highlighted in Yellow is a queried isolate.

Molecular identification through 16S rRNA gene sequencing demonstrated the presence of 600 bp DNA product (Figure 2), isolate SM7 exhibited 100.0% sequence similarity with *Streptomyces greisus* (Figure 3).

### 3.2 Antibacterial Activity Against ESBL‐Producing Bacteria

The crude ethyl acetate extract of *Streptomyces* sp. SM7 demonstrated significant antibacterial efficacy against all evaluated ESBL‐producing clinical isolates (Table 1).

**Table 1:** Antibacterial activity of *Streptomyces* sp. SM7 secondary metabolite extract against ESBL‐producing bacteria.

Test organism (ESBL‐producing)	Zone of inhibition (mm)—*Streptomyces* extract (100 µg/mL)	Zone of inhibition (mm)—Ciprofloxacin (5 µg)	*p*‐value vs. ciprofloxacin
*Escherichia coli*	21.4 ± 0.6	26.4 ± 0.5	0.00045
*Klebsiella pneumoniae*	19.2 ± 0.4	24.7 ± 0.6	0.00042
*Enterobacter cloacae*	17.6 ± 0.5	23.1 ± 0.4	0.00016

*Note:* Values are mean ± SD (*n* = 3). Exact *p*‐values are based on comparison with the positive control (ciprofloxacin) using triplicate summary statistics. No inhibition was observed with the solvent control.

**Figure 4:**
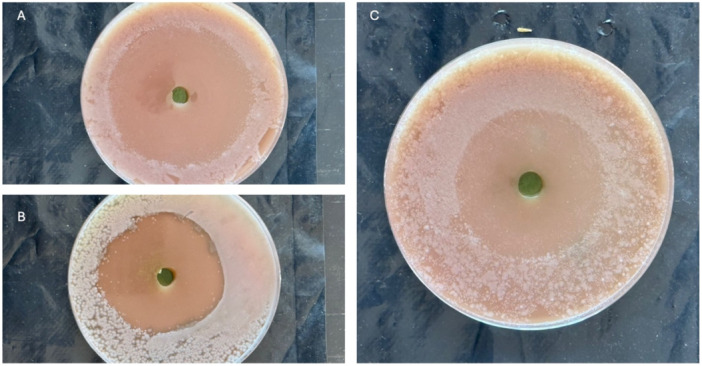
Antibacterial sensitivity test of the *Streptomyces* sp. SM7 extract on the test isolates. (A) *Escherichia coli Streptomyces* extract (100 µg/mL). (B) *Klebsiella pneumoniae Streptomyces* extract (100 µg/mL). (C) *Enterobacter cloacae Streptomyces* extract (100 µg/mL).

At a concentration of 100 µg/mL, the extract generated inhibition zones of 21.4 ± 0.6 mm for ESBL‐producing *Escherichia coli*, 19.2 ± 0.4 mm for *K. pneumoniae*, and 17.6 ± 0.5 mm for *Enterobacter cloacae*. The inhibition zones were analogous to those generated by ciprofloxacin, measuring between 23.1 and 26.4 mm (Figure 4).

The absence of inhibitory activity in the solvent control confirms that the antibacterial effects were due to the metabolites derived from *Streptomyces*.

### 3.3 Minimum Inhibitory and Bactericidal Concentrations

The broth microdilution assay demonstrated low MIC and MBC values for the crude extract against ESBL‐producing bacteria (Table 2).

**Table 2:** Minimum inhibitory concentration (MIC) and minimum bactericidal concentration (MBC) of *Streptomyces* sp. SM7 extract.

Test organism (ESBL‐producing)	MIC (µg/mL)	MBC (µg/mL)	MBC/MIC ratio	Mode of action
*Escherichia coli*	62.5	125	2	Bactericidal
*Klebsiella pneumoniae*	125	250	2	Bactericidal
*Enterobacter cloacae*	250	500	2	Bactericidal

*Note:* MBC/MIC ≤ 4 indicates bactericidal activity. In this study, MBC was defined as ≥ 99.9% killing of the starting inoculum; the ≤ 4 interpretation threshold follows the conventional bactericidal criterion.

The MIC values were 62.5 µg/mL for *E. coli*, 125 µg/mL for *K. pneumoniae*, and 250 µg/mL for Enterobacter cloacae. The corresponding MBC values were 125 µg/mL, 250 µg/mL, and 500 µg/mL, respectively. The MBC/MIC ratios (≤ 4) demonstrated a bactericidal mechanism of action for the extract.

### 3.4 Antioxidant Activity of *Streptomyces* Secondary Metabolites

#### 3.4.1 DPPH Radical Scavenging Activity

The crude extract of *Streptomyces* sp. SM7 exhibited potent, concentration‐dependent DPPH radical scavenging activity. At a concentration of 200 µg/mL, the extract demonstrated 82.6 ± 1.3% inhibition, whereas ascorbic acid exhibited 91.4 ± 0.8% inhibition at the identical concentration (Table 3).

**Table 3:** DPPH radical scavenging activity of *Streptomyces* sp. SM7 extract.

Concentration (µg/mL)	% Inhibition—*Streptomyces* extract	% Inhibition—Ascorbic acid	*p*‐value vs. ascorbic acid
25	28.7 ± 0.9	45.6 ± 1.1	0.00004
50	49.8 ± 1.2	68.9 ± 0.8	0.00006
100	66.3 ± 1.5	81.7 ± 0.9	0.00037
200	82.6 ± 1.3	91.4 ± 0.8	0.00136
IC₅₀ (µg/mL)	48.9	21.7	—

*Note:* Values are mean ± SD (*n* = 3). Exact *p*‐values are provided for comparison with ascorbic acid at matched concentrations.

The IC₅₀ value of the extract was determined to be 48.9 µg/mL, in contrast to 21.7 µg/mL for ascorbic acid, signifying considerable antioxidant efficacy.

#### 3.4.2 ABTS Radical Cation Scavenging Activity

The extract demonstrated 76.3% ± 1.1% inhibition in the ABTS assay at a concentration of 200 µg/mL. The IC₅₀ value was established at 61.4 µg/mL, whereas the IC₅₀ for ascorbic acid was recorded at 24.5 µg/mL (Table 4). The findings validated the extensive free radical scavenging capacity of the metabolites derived from *Streptomyces*.

**Table 4:** ABTS radical cation scavenging activity of *Streptomyces* sp. SM7 extract exact *p*‐values are provided for comparison with ascorbic acid at matched concentrations.

Concentration (µg/mL)	% Inhibition—*Streptomyces* extract	% Inhibition—Ascorbic acid	*p*‐value vs. ascorbic acid
25	24.6 ± 0.7	42.3 ± 0.9	0.00002
50	43.5 ± 1.1	64.8 ± 0.6	0.00007
100	61.9 ± 1.4	79.6 ± 0.8	0.00022
200	76.3 ± 1.1	90.2 ± 0.7	0.00016
IC₅₀ (µg/mL)	61.4	24.5	—

### 3.5 GC–MS Profiling of Bioactive Secondary Metabolites

The GC–MS analysis of the ethyl acetate extract from *Streptomyces* sp. SM7 identified 18 bioactive compounds, primarily consisting of phenolic compounds, fatty acid derivatives, and polyketide‐related molecules (Table 5). Because compound annotation was library‐based, these assignments should be interpreted as tentative pending confirmation with authentic standards.

**Table 5:** GC–MS identified bioactive compounds in *Streptomyces* sp. SM7 ethyl acetate extract.

Peak no.	Retention time (min)	Identified compound	Molecular formula	Molecular mass (g/mol)	Peak area (%)	Relative abundance (%)	Reported biological activity
1	9.34	2,4‐di‐tert‐butylphenol	C₁₄H₂₂O	206.32	18.9	18.6	Antibacterial, antioxidant
2	12.87	Hexadecanoic acid, methyl ester	C₁₇H₃₄O₂	270.45	14.6	14.2	Antibacterial, anti‐inflammatory
3	15.46	9,12‐Octadecadienoic acid (Z,Z)‐	C₁₈H₃₂O₂	280.45	12.1	11.7	Antioxidant, antimicrobial
4	17.02	Dibutyl phthalate	C₁₆H₂₂O₄	278.34	9.7	9.4	Antibacterial
5	18.76	Octadecanoic acid	C₁₈H₃₆O₂	284.48	8.2	7.9	Membrane disruption
6	20.11	1‐Hexadecanol	C₁₆H₃₄O	242.44	6.6	6.3	Antimicrobial

*Note:* Compound identification was performed by comparison with the NIST mass spectral library.

**Figure 5:**
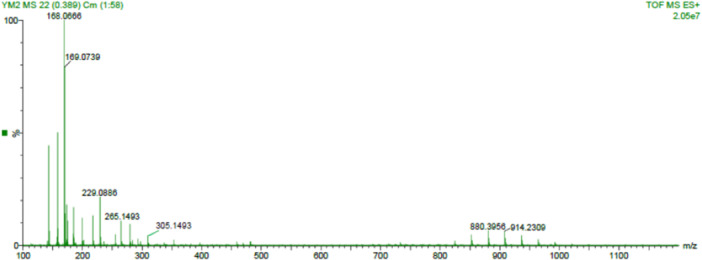
The GCMS spectra of *Streptomyces* sp. SM7 constituents.

Key identified compounds comprised 2,4‐di‐tert‐butylphenol (18.6%), hexadecanoic acid, methyl ester (14.2%), 9,12‐octadecadienoic acid (Z, Z)‐ (11.7%), and dibutyl phthalate (9.4%). The representative spectra is presented in Figure 5. Numerous compounds have been previously documented to exhibit antibacterial, antioxidant, and membrane‐disruptive properties. The presence of phenolic and fatty acid derivatives likely enhanced the observed antibacterial and antioxidant activities synergistically.

#### 3.5.1 Molecular Docking

Among the compounds identified by GC–MS, 2,4‐di‐tert‐butylphenol was chosen for molecular docking studies because of its advantageous molecular size, functional groups conducive to hydrogen bonding, established antimicrobial properties, and significant relevance to bacterial enzymatic targets such as DNA gyrase and dihydrofolate reductase. Details of the selected ligands and the protein receptors are provided in Table 6. This selection does not exclude a contributory role for the abundant fatty‐acid‐derived constituents; rather, it reflects the use of a mechanistically tractable lead compound for a first‐pass structure‐based analysis.

**Table 6:** Ligand and receptor identification for molecular docking studies ligand information.

Ligand code	Compound name	PubChem CID	Molecular formula	Molecular mass (g/mol)
L1	2,4‐di‐tert‐butylphenol	7311	C₁₄H₂₂O	206.32
Receptor (Protein Target) Information				
Receptor code	Target protein	Test organism	PDB ID	Biological relevance
R1	DNA gyrase subunit B	*Escherichia coli* (ESBL)	1KZN	Target for antibacterial inhibition
R2	Dihydrofolate reductase (DHFR)	*Escherichia coli* (ESBL)	1RX2	Folate metabolism inhibition
R3	DNA gyrase subunit B	*Klebsiella pneumoniae* (ESBL)	6RKS	Target for quinolone‐like inhibition
R4	Penicillin‐binding protein 2 (PBP2)	*Enterobacter cloacae*	3VMA	Cell wall biosynthesis

Table 7 encapsulates the anticipated binding affinities and critical interactions of 2,4‐di‐tert‐butylphenol (L1) with bacterial targets associated with ESBL‐mediated resistance. The Ligand demonstrated the most robust predicted interaction with *E. coli* DHFR (R2, Δ*G* = −7.1 kcal/mol), indicating potential inhibition of folate metabolism, an essential pathway for bacterial growth. The DNA gyrase targets (*E. coli* R1 and *K. pneumoniae* R3) exhibited moderate binding energies ( − 6.1 to −6.3 kcal/mol), predominantly stabilized by hydrophobic interactions within the ATP‐binding pocket.

**Table 7:** Predicted molecular docking scores of 2,4‐ditert‐butylphenol with target proteins.

Receptor code	Target protein (PDB ID)	Binding energy (Δ*G*, kcal/mol)	Key interacting residues	Modeled interaction notes
R1	*E. coli* DNA gyrase B (1KZN)	−6.1	Hydrophobic contacts	Moderate affinity toward the ATP‐binding pocket
R2	*E. coli* DHFR (1RX2)	−7.1	OH‐mediated H‐bonding	Favorable interactions with conserved active site residues
R3	*K. pneumoniae* DNA gyrase B (6RKS)	−6.3	Hydrophobic and π interactions	Similar motif to R1 due to conserved binding site

**Figure 6:**
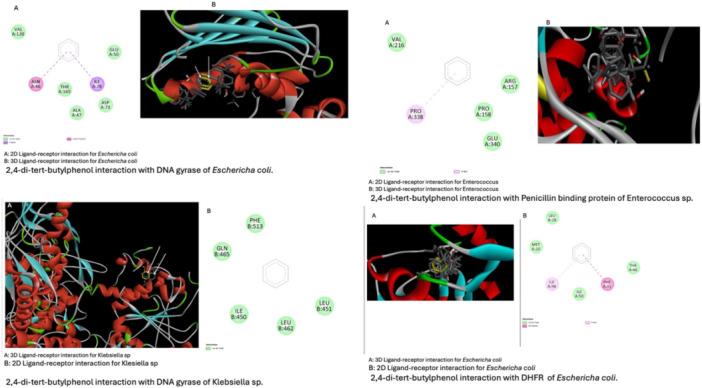
Ligand‐receptor interaction of a major compound derived from *Streptomyces* sp. SM7 against major bacteria proteins.

The results suggest that 2,4‐di‐tert‐butylphenol interacts with various bacterial targets, with its strongest affinity for DHFR, which aligns with a potential mechanism for antibacterial activity, corroborated by the observed in vitro inhibition of ESBL‐producing strains. The interplay of hydrogen bonding through the phenolic –OH group and hydrophobic interactions enhanced the anticipated docking stability, affirming the function of this metabolite as a bioactive constituent of *Streptomyces* sp. SM7 extracts. The docking results validate the experimental antibacterial findings and emphasize DHFR inhibition as the probable molecular mechanism responsible for the observed activity (Figure 6).

## 4 Discussion

The identification of new antimicrobial agents from soil‐derived *Streptomyces* remains a promising strategy for combating the increasing threat of multidrug‐resistant Gram‐negative pathogens, especially ESBL‐producing bacteria. The menace of antibiotic resistance is still a global concern (Zhuang et al. 2023), alternative sources of potent antibiotic have proven useful as new pharmacological intervention (Yang et al. 2025). This study selected *Streptomyces* sp. SM7 is due to its exceptional antibacterial activity observed in primary screening. Morphological and molecular analyses established its close association with *Streptomyces griseus*, a species historically acknowledged for synthesizing various bioactive metabolites, such as aminoglycosides and polyketides (Sardari 2017; Alam et al. 2022a). The elevated bioactivity of SM7 aligns with the observations of Zhang et al. (2020), who highlighted that the selective isolation of metabolically active strains increases the probability of discovering clinically relevant compounds (Zhang et al. 2020). Relative to recent *Streptomyces* reports from 2023 to 2025 (Chanama et al. 2023; Tfaily and Borjac 2025), SM7 is distinguished by the combination of confirmed activity against ESBL‐producing Enterobacterales, concurrent antioxidant activity, chemical profiling of the crude extract, and a target‐oriented docking analysis centered on a dominant phenolic constituent. Recent studies have described *Streptomyces* strains active against drug‐resistant bacteria or possessing strong antibacterial/antioxidant profiles, but many focused on broader MDR panels, non‐ESBL pathogens, or taxonomic description rather than integrating anti‐ESBL bioassays with metabolite‐level interpretation (Rammali et al. 2024; Potapenko et al. 2025).

The crude ethyl acetate extract of SM7 exhibited significant antibacterial activity against all evaluated ESBL‐producing strains. The inhibition zones observed against *E. coli, K. pneumoniae*, and *E. cloacae* were significant, nearing the effectiveness of ciprofloxacin. These findings corroborate earlier reports demonstrating that metabolites from *Streptomyces* possess broad‐spectrum efficacy against Gram‐negative pathogens, including those resistant to β‐lactam antibiotics (Khaledi and Obeid 2025; Benhadj et al. 2020). The extract's bactericidal properties, indicated by low MIC and MBC values with MBC/MIC ratios ≤ 4, imply that the secondary metabolites inhibit critical bacterial functions directly rather than solely restricting growth. This mechanism aligns with research on other phenolic and fatty acid compounds derived from *Streptomyces*, which frequently exhibit irreversible bactericidal effects via membrane disruption and enzyme inhibition (Sanjivkumar et al. 2016; Abdulkareem et al. 2024).

The extract's chemical composition, as indicated by GC–MS, demonstrates a predominance of phenolic compounds, fatty acid derivatives, and polyketide‐related molecules. Significantly, 2,4‐di‐tert‐butylphenol constituted the predominant component, accounting for 18.6% of the extract, followed by hexadecanoic acid methyl ester at 14.2% and 9,12‐octadecadienoic acid at 11.7%. Phenolic compounds are well‐known for their capacity to disrupt bacterial cell membranes, inhibit essential enzymatic functions, and destabilize cellular homeostasis (Yang et al. 2024). Fatty acid derivatives enhance antibacterial activity by engaging in hydrophobic interactions with bacterial lipid bilayers, potentially compromising membrane integrity and allowing the ingress of other bioactive metabolites (Obukhova and Murzina 2024). The coexistence of these metabolite classes likely underlies the extensive activity observed against ESBL‐producing strains, illustrating the principle of synergistic interaction among secondary metabolites in *Streptomyces* extracts (Alam et al. 2022b). Although hexadecanoic acid methyl ester was also abundant, the present docking work focused on 2,4‐di‐tert‐butylphenol because its phenolic hydroxyl group and compact aromatic framework offer a clearer basis for enzyme‐target interaction analysis than long‐chain ester derivatives, which may instead contribute through less specific membrane‐disruptive effects.

Molecular docking analysis offered further mechanistic understanding of the antibacterial efficacy of SM7 metabolites. 2,4‐di‐tert‐butylphenol demonstrated the highest anticipated binding affinity for *E. coli* dihydrofolate reductase (DHFR, Δ*G* = −7.1 kcal/mol), suggesting that the inhibition of folate metabolism is a principal mechanism of antibacterial activity. The binding affinity to DNA gyrase was moderate (Δ*G* = −6.1 to −6.3 kcal/mol), suggesting alternative mechanisms of disruption in bacterial DNA replication. The findings align with those of Elsewedy et al. (2024 and Spencer and Panda (2023), who indicated that small phenolic compounds frequently interact with multiple bacterial enzymes, thereby broadening their antibacterial spectrum and reducing the potential for resistance emergence. The hydrogen bonding from the phenolic –OH group, along with hydrophobic interactions from tert‐butyl substituents, likely stabilized these interactions, thereby supporting the observed in vitro bactericidal activity. This multi‐target interaction pattern is especially pertinent for addressing ESBL‐producing strains, which frequently exhibit enzyme‐mediated resistance mechanisms that make single‐target antibiotics ineffective. The docking results should therefore be interpreted as hypothesis‐generating rather than definitive proof of target engagement, especially because only one constituent was docked.

The extract's efficacy against ESBL‐producing bacteria is significant, as these pathogens pose clinical challenges due to their capacity to hydrolyse broad‐spectrum β‐lactams. The capacity of SM7 metabolites to impede growth suggests that their mechanism of action may bypass traditional resistance pathways, potentially by targeting DHFR, enzymes involved in cell wall synthesis, and membrane structures. Similar research has shown that phenolic and fatty acid derivatives from *Streptomyces* can impede multidrug‐resistant Enterobacteriaceae by compromising membranes and inhibiting critical enzymes (Rocha et al. 2025; Shamshad et al. 2022; Peraman et al. 2021). The demonstrated bactericidal effects of SM7 metabolites against clinically significant ESBL strains underscore the promise of *Streptomyces* secondary metabolites as alternative or supplementary therapeutic agents.

The extract demonstrated significant antibacterial activity and robust antioxidant potential in both DPPH and ABTS assays, with IC₅₀ values of 48.9 and 61.4 µg/mL, respectively. While antioxidant activity does not directly influence antibacterial effects, it can mitigate oxidative stress in host tissues during infection, bolster cellular resilience, and diminish the accumulation of pathogen‐induced reactive oxygen species (ROS) (Edge and Truscott 2021). The combined antibacterial and antioxidant properties align with findings on other *Streptomyces* metabolites, which have demonstrated therapeutic advantages through direct microbial inhibition and reduction of oxidative stress (Fouda et al. 2020). The pronounced free radical scavenging activity exhibited by SM7 metabolites may also improve the stability and efficacy of phenolic compounds, which are frequently vulnerable to oxidative degradation, thereby promoting prolonged antibacterial activity. No direct synergy experiments were performed in the present study; accordingly, any therapeutic interaction between antibacterial and antioxidant effects should be regarded as a hypothesis for future work rather than an established conclusion.

The integration of chemical profiling, in vitro activity, and in silico docking offers a comprehensive rationale for the demonstrated antibacterial efficacy. Phenolic compounds, including 2,4‐di‐tert‐butylphenol, are likely the primary bioactive agents, whereas fatty acids and minor polyketides provide supplementary or synergistic effects. The docking results further confirm that DHFR inhibition is a probable mechanism, reinforced by hydrogen bonding and hydrophobic interactions, which align closely with the experimentally observed bactericidal effects. The results align with the studies by Ait Assou & El Hassouni et al. (2025) and Pattapulavar et al. (2025), which documented analogous synergistic antibacterial mechanisms in mixtures of metabolites derived from *Streptomyces*. Despite the efforts for new detection techniques associated to bacterial sepsis (Liu et al. 2022), antibiotic resistance war is still very active in the current century.

This study illustrates that *Streptomyces* sp. SM7 generates a varied array of bioactive secondary metabolites that can inhibit clinically relevant, multidrug‐resistant ESBL‐producing bacteria. The amalgamation of GC–MS profiling and molecular docking enhances mechanistic comprehension and is consistent with previous literature regarding the multi‐target efficacy of phenolic‐rich *Streptomyces* extracts (Sebak et al. 2025; Abbas et al. 2025). The results underscore the therapeutic promise of these metabolites, stressing the significance of natural product‐oriented approaches in addressing the worldwide challenge of antimicrobial resistance.

## 5 Conclusion

*Streptomyces* sp. SM7 synthesizes secondary metabolites exhibiting significant bactericidal efficacy against ESBL‐producing *E. coli, K. pneumoniae*, and *E. cloacae*. GC–MS profiling identified 2,4‐di‐tert‐butylphenol and fatty acid derivatives as primary bioactive compounds, potentially facilitating membrane disruption and enzyme inhibition. The extract demonstrated significant antioxidant activity, reinforcing its dual bioactivity. Molecular docking revealed that DHFR inhibition is a principal mechanism, accompanied by supplementary interactions with DNA gyrase. These results support continued bioassay‐guided purification and preclinical evaluation of SM7 metabolites as prospective adjuncts or leads for the treatment of infections caused by drug‐resistant Enterobacterales.

## Author Contributions

**Abdirasak Sharif Ali:** conceptualization, investigation, funding acquisition, writing – original draft, methodology. **Yahye Ahmed Nageye:** conceptualization, methodology, visualization, supervision, writing and editing of the manuscript. **Kizito Eneye Bello:** validation, visualization, supervision.

## Ethics Statement

Clinical bacterial isolates were retrieved from anonymized laboratory collections in accordance with applicable institutional procedures; no human subjects were directly enrolled, and no patient‐identifying information was used.

## Conflicts of Interest

The authors declare no conflicts of interest.

## Supporting information


**Supplementary Table 1:** Preliminary Screening of *Streptomyces* Isolates (SM1–SM8) Against ESBL‐Producing Bacteria.
