# Supplementary material for: Antioxidant and Antibacterial Activities of Secondary Metabolites Produced by Streptomyces Isolates Against Extended‐Spectrum β‐Lactamase‐Producing Bacteria
Source: Microbiologyopen. 2026 Apr 19;15(2):e70282. doi: 10.1002/mbo3.70282 (PMC13092211; doi:10.1002/mbo3.70282)
Supplement: Supplementary file 1 — Supplementary Table 1: Preliminary Screening of Streptomyces Isolates (SM1–SM8) Against ESBL‐Producing Bacteria. [file MBO3-15-e70282-s001.docx]

Supplementary Table 1. Preliminary Screening of Streptomyces Isolates (SM1–SM8) Against ESBL-Producing Bacteria

| Streptomyces isolate | *E. coli* (mm) | *K. pneumoniae* (mm) | *E. cloacae* (mm) | Mean inhibition zone (mm) |
| --- | --- | --- | --- | --- |
| SM1 | 10.2 ± 0.5 | 9.8 ± 0.6 | 8.9 ± 0.4 | 9.6 |
| SM2 | 11.4 ± 0.4 | 10.6 ± 0.5 | 9.7 ± 0.5 | 10.6 |
| SM3 | 12.1 ± 0.6 | 11.3 ± 0.4 | 10.4 ± 0.5 | 11.3 |
| SM4 | 13.0 ± 0.5 | 12.2 ± 0.6 | 11.1 ± 0.4 | 12.1 |
| SM5 | 14.2 ± 0.4 | 13.5 ± 0.5 | 12.6 ± 0.6 | 13.4 |
| SM6 | 15.6 ± 0.5 | 14.7 ± 0.4 | 13.9 ± 0.5 | 14.7 |
| **SM7** | **21.4 ± 0.6** | **19.2 ± 0.4** | **17.6 ± 0.5** | **19.4** |
| SM8 | 13.7 ± 0.6 | 12.8 ± 0.5 | 11.9 ± 0.4 | 12.8 |

Values represent mean zone of inhibition ± SD (mm) from triplicate experiments (n = 3). Crude secondary metabolite extracts from eight presumptive *Streptomyces* isolates were screened against ESBL-producing bacterial strains using the agar diffusion method. Isolate SM7 exhibited the highest antibacterial activity and was selected for subsequent characterization and detailed activity analysis.
